# Adapting a health facility HIV stigma-reduction participatory training intervention to address drug use stigma in HIV care and treatment clinics in Dar es Salaam, Tanzania

**DOI:** 10.1186/s12954-024-00965-4

**Published:** 2024-03-15

**Authors:** Linda B. Mlunde, Khalida Saalim, Jessie K. Mbwambo, Pfiriael Kiwia, Elizabeth Fitch, Willbrord Manyama, Isack Rugemalila, Sue Clay, Barrot H. Lambdin, Rachel D. Stelmach, Carla Bann, Laura Nyblade

**Affiliations:** 1 Muhimbili University of Health and Allied Sciences, Dar es Salaam, Tanzania; 2 RTI International, Washington, D.C. USA; 3 Muhimbili National Hospital, Dar es Salaam, Tanzania; 4 Kimara Peer Educators and Health Promoters, Dar es Salaam, Tanzania; 5 RTI International, Research Triangle Park, NC, USA; 6 Temeke Regional Referral Hospital, Dar es Salaam, Tanzania; 7 3C Regional Consultants, Lusaka, Zambia; 8 RTI International, Berkley, CA USA

**Keywords:** Tanzania, Drug abuse, Substance use, Social stigma, Stereotyping, Health facilities, Curriculum

## Abstract

**Background:**

HIV prevalence among people who use drugs (PWUD) in Tanzania is 4–7 times higher than in the general population, underscoring an urgent need to increase HIV testing and treatment among PWUD. Drug use stigma within HIV clinics is a barrier to HIV treatment for PWUD, yet few interventions to address HIV-clinic drug use stigma exist. Guided by the ADAPT-ITT model, we adapted the participatory training curriculum of the evidence-based Health Policy Plus Total Facility Approach to HIV stigma reduction, to address drug use stigma in HIV care and treatment clinics (CTCs).

**Methods:**

The first step in the training curriculum adaptation process was formative research. We conducted 32 in-depth interviews in Dar es Salaam, Tanzania: 18 (11 men and 7 women) with PWUD living with HIV, and 14 with a mix of clinical [7] and non-clinical [7] CTC staff (5 men and 9 women). Data were analyzed through rapid qualitative analysis to inform initial curriculum adaptation. This initial draft curriculum was then further adapted and refined through multiple iterative steps of review, feedback and revision including a 2-day stakeholder workshop and external expert review.

**Results:**

Four CTC drug use stigma drivers emerged as key to address in the curriculum adaptation: (1) Lack of awareness of the manifestations and consequences of drug use stigma in CTCs (e.g., name calling, ignoring PWUD and denial of care); (2) Negative stereotypes (e.g., all PWUD are thieves, dangerous); (3) Fear of providing services to PWUD, and; (4) Lack of knowledge about drug use as a medical condition and absence of skills to care for PWUD. Five, 2.5-hour participatory training sessions were developed with topics focused on creating awareness of stigma and its consequences, understanding and addressing stereotypes and fears of interacting with PWUD; understanding drug use, addiction, and co-occurring conditions; deepening understanding of drug use stigma and creating empathy, including a panel session with people who had used drugs; and working to create actionable change.

**Conclusion:**

Understanding context specific drivers and manifestations of drug use stigma from the perspective of PWUD and health workers allowed for ready adaptation of an existing evidence-based HIV-stigma reduction intervention to address drug use stigma in HIV care and treatment clinics. Future steps include a pilot test of the adapted intervention.

## Background

In 2020, 275 million people were reported to use drugs worldwide [1]. Across the African continent, it is estimated that 60 million people aged 15–64 years used drugs at least once in the past year [1]. Projections show that drug use will increase across the globe, with an increase of 40% in Africa by the year 2030 [1]. The United Republic of Tanzania experiences a high and growing burden of drug use [2]. The most recent estimates, which are from 2014, were that 300,000 people were using drugs in the country, primarily heroin, with some polysubstance use involving cocaine, cannabis, tobacco, and khat [3]. 10% (30,000: range 20,000–42,500) of people who use drugs (PWUD) were injecting [2–5]. In addition, PWUD in Tanzania, both those who inject drugs and those who do not, are at a high risk for HIV acquisition. In Tanzania, HIV prevalence among PWUD who inject (35%) and PWUD who do not inject (18–25%) is 4–7 times higher than in the general population (5%) [3, 6]. Despite this burden, access to HIV testing and treatment falls short among PWUD [7]. Many PWUD experience low uptake of HIV testing, anti-retroviral therapy (ART) initiation, and retention in care [8, 9].

Poor access to HIV care for PWUD results from various challenges they encounter, including stigma [10–12]. Stigma is a social process occurring in the context of power. It results in social disqualification based on real or perceived characteristics (e.g. a health condition or identity) and can lead to exclusion, rejection, blame, devaluation and discrimination [13]. Stigma has been found to be a barrier to HIV care and treatment services access around the world [14–16]. Globally, PWUD are highly stigmatized, with several studies documenting higher levels of stigma toward PWUD than people with other stigmatized health conditions [17–21]. Drivers of drug use stigma include fear (e.g., PWUD are dangerous, steal), causal attribution-beliefs that drug use is under personal control (e.g., PWUD are “too lazy to change”), and belief that drug use is a “moral failing” not a medical condition [22, 23]. Drug use stigma, including experienced, anticipated, perceived, and internalized, has been documented as a barrier to linkage and retention in medically assisted therapy (MAT) [24], needle and syringe exchange programs [25], HIV care and treatment services [10–12], and general health care [10, 26–31] and is negatively associated with physical and mental health outcomes among PWUD [32–34].

Stigma in health facilities poses a specific barrier to uptake of health services including HIV services [22, 35, 36] and health facilities can be a prominent source of stigma for PWUD [37]. Stigma experienced in the health facility carries a particular danger because it directly influences whether an individual will access care for their condition [38]. Within health facilities globally, documented manifestations of stigma include denial of care, lower quality of care, physical and verbal abuse, and longer wait times for those with stigmatized conditions [38]. With Tanzania not being an exception, PWUD have described similar experiences in health facilities [7], including in CTCs [39, 40]. They have also reported how that experience of stigma influenced risk of shying away from health care settings when in need of care [7]. Drug use stigma enacted by providers is reported as a barrier specifically to initiation of ART and continuation of care among PWUD in Tanzania [39, 40]. Recognition of the barrier stigma poses for HIV prevention and treatment, including for PWUD and the importance of addressing stigma for an effective national HIV response, is underscored by the National AIDS Control Programme’s most recent Health Sector HIV Strategic Plan V (2021–2026), which includes a focus on addressing stigma and discrimination particularly towards key and vulnerable populations [41].

Therefore, reducing drug use stigma in health services is an important strategy to improve access to and retention in health care for PWUD, including for HIV services [27]. However, the literature on tested drug use stigma-reduction interventions is still nascent. A 2012 review on effectiveness of interventions for reducing substance use disorder stigma identified only 13 studies, five of which focused on medical students, however none were with health facility staff or focused on HIV services [42]. Studies published since the review have been mostly in high-income countries and often delivered online [43, 44] or focused more broadly on key population stigma and not specifically on drug use stigma [45]. Therefore, for low-middle income countries, like Tanzania, that are responding to the dual challenges of HIV and substance use, understanding what is driving drug use stigma in HIV prevention and treatment services and how to apply this knowledge to appropriately adapt existing HIV stigma reduction interventions is needed.

In response, we adapted the participatory training curriculum of an evidence-based HIV stigma reduction intervention for health facilities (The Health Policy Plus (HP+) intervention) [46, 47] to specifically address drug use stigma in HIV care and treatment clinics (CTCs) in Dar es Salaam, Tanzania. The HP + HIV stigma-reduction training, originally developed in Tanzania and Ghana, is a manualized modular curriculum consisting of 10–14 h of participatory exercises covering key drivers of HIV stigma and is available in both English and Swahili. The modular curriculum allows for flexible timing in delivery of the curriculum, based on a health facility’s scheduling preference. Health facility staff and community members living with HIV receive a five-day training in participatory training methods and the stigma-reduction content to become stigma-reduction trainers. They then deliver the training to health facility staff [46]. The HP + training curriculum addresses three key drivers of facility HIV stigma through participatory methodologies: lack of awareness of stigma, fear of HIV acquisition, and stigmatizing attitudes rooted in stereotypes and misconceptions. Based in social cognitive theory and interpersonal or intergroup contact theory it fosters empathy and builds efficacy for stigma reduction through building awareness and knowledge, as well as skills to change individual behavior and work collectively to alter the health facility environment [46].

In this article, we present the process and results of the first phase of the adaptation of the HP + HIV stigma reduction training curriculum to address drug use stigma in CTCs in Tanzania. We share the key drivers and manifestations of drug use stigma that emerged from the formative qualitative research which informed and shaped the initial step in the adaptation of the HP + HIV stigma-reduction curriculum. We then describe how these drivers, as well as clinic drug use stigma manifestations, are addressed in the curriculum. Understanding the drivers and manifestations of drug use stigma in HIV CTCs and the process of shaping the content of the curriculum to respond to these drivers not only offers important process data on how to adapt stigma reduction interventions but is also essential to allow for replicability in other settings. The adapted drug use stigma reduction curriculum is undergoing pilot testing in the second phase of the study.

## Methods

We employed a modified version of the ADAPT-ITT model [48], a standard method for adapting HIV-related evidence-based interventions. To arrive at a final adapted curriculum ready for pilot-testing, we implemented the first six steps of the ADAPT-ITT model. The final step of the model, the pilot testing, is ongoing.

### Step 1: assess: formative research

The first step in our adaptation process was conducting qualitative in-depth interviews (IDIs) in February 2021 with both CTC staff and PWUD living with HIV. The aim of this initial step was to inform the adaptation of the HP + HIV stigma-reduction training curriculum to focus on drug use stigma-reduction. The study team developed the interview guides for PWUD and for CTC staff based on their experience in stigma research and intervention adaptation, HIV service delivery to PWUD and the literature. The interview guides for PWUD consisted of questions which sought to understand the types of stigma (experienced, anticipated, and perceived) and their manifestations that PWUD encounter in relation to CTCs, how this stigma influences HIV care linkage and retention, and drivers of drug use stigma in CTCs. The interview guide for CTC staff had questions that sought to understand the same issues but in addition, we asked them about their experiences of providing services to PWUD. The interview guides were developed in English by the core research team through an iterative process of development, team discussion and revision. This team included multiple bi-lingual (Swahili and English) study team members, including the principal investigator (PI) and site PI. The guides were then translated by a translator who was not part of the research team into Swahili. The Swahili was then back translated by a different independent translator into English and reviewed to ensure that the intent of the original guiding questions and probes in English was retained. The interview guides were pilot tested in Swahili through role plays during the research assistants training before data collection. Minor revisions were made on the interview guides after the pilot testing.

#### Participant selection and recruitment

Health facility staff were recruited from seven study CTCs located in areas of Dar es Salaam with the highest concentration of PWUD—Ilala, Kinondoni, and Temeke Municipal Councils and surrounding communities. Study clinics were selected through a two-step process. We mapped each CTC’s distance from a high drug use area based on existing data. We randomly selected seven facilities out of the list of all CTCs that would provide close and easy access to the largest concentrations of PWUD (within 5 km).

We interviewed both clinical staff who provide clinical services to PWUD and other clients, including doctors, nurses, nutritionist, phlebotomists and pharmacists (*n* = 7; 5 female/2 male) and non-clinical who provide support services in health facilities, e.g. cleaners, security guards, data officers and peers (*n* = 7; 4 female/3 male) CTC staff aged 18 or older who had direct contact with clients seeking HIV treatment services. Two CTC staff (1 clinical/1 non-clinical) were randomly recruited from each of the seven study CTCs for a total of 14 CTC staff.

PWUD living with HIV (*n* = 18) were purposively sampled with the assistance of community-based organizations (CBOs) providing services for PWUD using heroin in the communities surrounding the study clinics. To gather a range of experiences, we interviewed PWUD living with HIV in the following categories: five (2 males/3 females) participants on MAT who were also receiving HIV treatment at a CTC; four (3 males/1 female) MAT clients not on HIV treatment; nine (6 males/3 females) participants currently using heroin who were not receiving MAT or HIV treatment. To be eligible for the study, PWUD had to be living with HIV, be a resident of Dar es Salaam for at least the past three months, be 18 years or older and mentally competent to participate in an interview.

#### Data collection

Three experienced research assistants and a study coordinator conducted the IDIs. They received a three-day training on the study protocol, procedures, ethics, stigma, and qualitative methods. IDIs were conducted in Swahili in a private location using a semi-structured interview guide. After each interview, the interviewers filled in a debrief form to summarize the interview and main findings. A debrief meeting was also held with the Tanzania site Principal Investigator at the end of each day of data collection. Interviewers recorded the interviews and transcribed the recordings verbatim. The Tanzania site Principal Investigator and study coordinator checked the transcripts for quality.

#### Data analysis

All transcripts were translated from Swahili to English. We then conducted a rapid qualitative analysis (RQA). RQA is an increasingly common analysis method used in health services research to rapidly aggregate findings to inform practice and policy [49–53]. The study team jointly created a template to rapidly extract findings from transcripts, which was used to develop summary memos for each of the transcripts, containing major themes found in each transcript. Four researchers conducted the analysis and created summary memos for their assigned transcripts. Each transcript was reviewed by only one researcher who then developed a summary. Once the summary memos were completed, the full study team read all the summaries and met to discuss them and agree on key themes for intervention adaptation. These were combined into a single summary memo describing the drivers and manifestations of drug use stigma towards PWUD, which informed the next step.

### Step 2: decision

In this step, the study team, together with master stigma-reduction trainers from study partner Kimara Peer Educators and Training Trust (Kimara Peers)—a CBO with twenty years of experience delivering stigma-reduction interventions–conducted the initial adaptation of the HP + HIV stigma-reduction curriculum through virtual and in-person working sessions. The team was guided by the RQA results and the study team’s collective experience working with PWUD through delivering outreach, MAT, and HIV services and developing and adapting HIV and related stigma-reduction intervention tools for a range of audiences [54–58].

### Step 3: administer

Next, a review of the first draft of the adapted training materials was conducted through a 2-day participatory stakeholder workshop, led by Kimara Peers. Twenty people participated including CTC staff, people with lived experience of drug use, CBO staff providing services to PWUD, and municipality and ministry of health representatives. We asked workshop participants for feedback on their overall perceptions and experience of the training and to comment on the approach, length and content of each specific exercise, including relevance and appropriateness (including any visual materials). Participants liked the delivery modality and content of the curriculum, but felt sessions were too long and recommended they not run over 3 h.

### Step 4: produce

The study team then reviewed the stakeholder workshop feedback. This was followed by refining the training manual in response to the feedback. This included shortening the overall time of each module while ensuring the goals of those exercises were still attained.

### Step 5: topical experts

Two HIV stigma-reduction training experts and one drug use expert reviewed the adapted manual to provide feedback on its congruence with stigma-reduction training principles, the scientific literature, and observed experience of PWUD accessing HIV treatment. Experts included co-developers of the original HIV stigma-reduction toolkit [55] and of the first adaptation for health workers [57]; and one health care worker with extensive service delivery experience for PWUD in Tanzania. Topical experts confirmed congruence and provided minor suggestions on further refining specific exercises paying attention to timing, potential combining of exercises, role plays, visual materials (e.g., pictures) and process.

### Step 6: integrate

The study team conducted a final revision of the manual incorporating feedback from the topical experts.

## Ethical considerations

The study was approved by the Muhimbili University of Health and Allied Sciences (MUHAS) Senate Research and Publications Committee (MUHAS-REC-11-2020-416) and National Review Ethics Committee (NatHREC) of National Institute of Medical Research (NIMR) (NIMR/HQ/R.8a/Vol.IX/3556). Permission to conduct the study was obtained from the municipal councils and health facilities. Considering the illegality of drug use in Tanzania, oral informed consent was obtained from PWUD. This approach is considered the most ethical when conducting interviews with highly stigmatized populations who engage in criminalized behavior [59]. Written informed consent was collected from CTC staff before data collection.

## Results

We present first the key themes that emerged from the formative research, specifically the key drivers of drug use stigma and associated stigma manifestations in CTCs, as well as the consequences of drug use stigma for HIV care and treatment. We then describe how we addressed the key drivers and manifestations through the adapted curriculum.

### Key actionable drivers, associated stigma manifestations and consequences of drug use stigma

Four drivers and associated manifestations emerged from the formative research as key themes the drug use stigma reduction training should address.

#### 1) Lack of awareness of the manifestations and consequences of drug use stigma in HIV clinics

Language is a means of communication; however, one may not be aware that the words that he/she is using to communicate are stigmatizing. Non-recognition, or lack of awareness of stigmatizing language with clients who use drugs or stigmatizing behaviors, like gossiping, emerged as a driver of drug use stigma in the CTCs. Both health facility staff (HFS) and PWUD participants reported that PWUD are often called by stigmatizing and hurtful names in the health facility, including ‘teja’ (junkie), ‘mwizi’ (thief), ‘wa kubembea’ (swinging), ‘mvuta unga’ (powder smoker) etc. However, some HFS did not realize that these names were felt as stigmatizing. One HFS even stated that calling PWUD these names was good as it would make PWUD stop using drugs. As he explained:

“The name is bad, but if you call a PWUD ‘teja’ (junkie), the name will be suitable because they will feel hurt then they will stop using drugs, because if anything hurts you, you will stop it.” (Nonclinical, Male)

HFS noted that gossiping about clients who use drugs with their colleagues and pointing fingers at them was commonplace. As explained:

“You point at them or talk about them aside… ‘do you see them, they are using drugs’.” (Clinical, Female)

While lack of awareness emerged as a driver of stigma in the data simply through HFS participants discussing stigmatizing language and actions without recognizing them as stigmatizing, a few HFS did recognize the lack of understanding and knowledge of stigma as a cause of stigma.

“Stigma happens [because] I think we also lack education…That education about stigma…or even those who use drugs because they too need that we give them a chance to listen to them.” (Clinical, Female)

#### 2) Negative stereotypes

Both HFS and PWUD reported on several commonly held stereotypes by HFS that paint clients who use drugs as aggressive, thieves, unclean and vectors of disease. These beliefs drove common stigmatizing actions and use of stigmatizing language towards clients who use drugs in the health service delivery setting, including CTCs.

For example, HFS expressed hesitancy in approaching or touching PWUD because of the perception that they are both physically dirty and spread diseases such as tuberculosis (TB) and HIV. PWUD participants confirmed this, reporting that HFS are afraid to touch them because they fear PWUD may transmit a disease to them. This results in HFS taking additional precautionary measures when providing services that are not taken with other clients, and so are experienced as stigmatizing by clients who use drugs while also marking PWUD as different from other clients. For example, one male PWUD participant described experiencing a HFS toss their medicines at them, instead of handing it to them, so as to not touch them. At the same time PWUD recognize that they are at increased risk for certain medical conditions and this drives the HFS behavior. As this participant explained further:

“…in my opinion I think it’s the fear of TB infection…because most people who use drugs are found with TB…we are found with TB…that’s why they are doing that to us.” (PWUD, Male)

#### 3) Fear of providing services to PWUD

In addition to fear of acquiring an infection from clients who use drugs, fear driven by the stereotypes that PWUD are aggressive and come to the health facility to steal to support their addiction leads many HFS to anticipate and fear being robbed or hurt by PWUD. As explained by a male HFS participant:

“At times we fear that may be when you’re at the counseling room they may harm you…That’s the biggest fear…We fear because we know some of people who are using drugs are aggressive…So we fear they can harm you. We all have that fear.” (Clinical, Male)

As explained by both HFS and PWUD, these fears led HFS to take stigmatizing actions of identifying PWUD in the facility, putting them under surveillance and taking extra safety precautions around them. For example, HFS frequently mentioned their safety concerns when encountering PWUD and how they took additional protective measures, like not wearing valuable items to prevent anticipated ‘attacks’ from PWUD. Fear of theft led to PWUD being overly questioned, surveilled, or followed by guards as a measure to ‘prevent them from stealing’.

“When a PWUD shows up at the gate, I resolve to following him on their way, if they go this way, I go that way.” (Nonclinical, Female)

“It depends if I work on those clinics where people who use drugs are. I will keep myself safe by not having those expensive things around me that can tempt that person to attack me.” (Clinical, Female)

#### 4) Lack of knowledge about drug use as a medical condition and absence of skills to care for PWUD

HFS demonstrated various misconceptions about addiction, including the belief that addiction is not a sickness and PWUD could easily choose to quit using drugs on their own volition. This, in turn facilitated the blame of PWUD for their condition and sometimes the dehumanization of PWUD, as it was difficult for HFS to see PWUD as a regular health client, suffering with a medical condition.

“It is just the understanding because one knows ‘aah this one [client] uses drugs, they aren’t sick.’ Or they have just come here, probably they pretend to be sick, but they want to steal from us.” (Clinical, Female)

Lack of skills on how to care for PWUD was also found to be a driver of drug use stigma. HFS worried that they would not be able to provide adequate care to PWUD when they visit the health facility, especially when they are under the influence of drugs. HFS feared interactions between drugs used by PWUD and medication they receive for treatment.

“You know this one [PWUD], if I give them this medicine and they are using drugs it may bring problems to them. Therefore I [a medical provider] hesitate in providing service too, so at the end of the day I may not attend them [PWUD] even for that emergency they have…Therefore, they have already missed that right of treatment.” (Clinical, Female)

Many clinical health workers felt too unprepared to provide health services to PWUD. They viewed drug use as a special condition requiring special management. Several HFS wanted more training to be able to treat PWUD.

“My other advice is that training [concerning people who use drugs] should be provided to healthcare providers…When we get training, it means if such a person comes here, we can manage him and we can take care of him as it is supposed to be done.” (Clinical, Female)

### Consequences

The overall results of these drivers and associated manifestations of drug use stigma are that PWUD often face poor quality of care at health facilities, including being subjected to longer waiting times than others and sometimes being denied health services outright. Some HFS do not take the health needs and concerns of PWUD seriously and believe that PWUD are only at the health facility to ask for money or drugs to support their addiction. Therefore, their time is not respected or valued by HFS. HFS described observing PWUD being ignored by colleagues while the needs of other clients were prioritized.

“A PWUD may be sitting here, next to them might be another patient who is not using drugs. A health worker may come and take the patient who is not using drugs and ignore the patient who is using drugs while both have the right to receive services.” (Clinical, Female)

One PWUD participant described a situation where they were completely ignored by HFS during an urgent and timely health situation.

“There is a time when I went to the hospital when I was ill. I was very ill. When I arrived there, I sat on the bench. Every doctor I called just looked at me and passed by. I was hurt by their reaction. I thought… maybe because I do not have money, I use drugs, so I am disrespected. It is better if I leave, as I did not get any assistance.” (PWUD, Male)

### How the adapted curriculum (*understanding and challenging drug use stigma in HIV care and treatment clinics*) responds to the drivers and associated manifestations of clinic drug use stigma

We addressed the key drivers and associated manifestations of drug use stigma by adapting eight of the original 16 exercises of the HP + HIV stigma reduction curriculum and by creating six new exercises to cover a key driver of drug use stigma that required new material. This led to a drug use stigma reduction training curriculum consisting of five, 2.5 to 3-hour training sessions with a total of 14 participatory exercises ranging from 20 to 90 min each, that could be delivered flexibly across five days of training (Table 1). Each of the drivers and various manifestations of drug use stigma that arose in the formative research were addressed across more than one session and in multiple exercises. The delivery of the training can differ from facility to facility based on the availability of staff and their preferred time to receive the training.

Decisions on which existing HP + participatory training exercises to adapt and which to discard was done through an assessment of the combination of each exercise’s appropriateness in terms of training modality (e.g., discussion, brainstorming, role play, reflection), whether the specific content was readily adjustable (e.g., case studies, pictures, drivers of stigma) to focus on drug use stigma as opposed to HIV stigma and most importantly addressed a key driver of drug use stigma. The amount of required adaptation varied from very little (for example the panel discussion with people of lived experience) to substantial, for example keeping the form and modalities of the exercise but creating all new case studies or role plays. Or in the case of the original exercise that addressed fear of HIV transmission as a driver of HIV stigma, adapting the exercise to address the specific fears of providing services to PWUD. Once this initial assessment of existing exercises was completed, we reviewed the set for completeness against the key drivers and their related stigma manifestations to assess gaps that required development of new materials. Specifically, we were missing exercises that addressed the driver of lack of knowledge about drug use as a medical condition and absence of skills to care for PWUD. We created six new exercises to fill this gap: all of the exercises in Session 3, exercises 2 and 3 in Session 2 and exercise 4 in Session 1. Throughout the adaptation, whether in tweaking existing exercises or creating new ones, we sought to build awareness and knowledge, foster empathy, reduce distance, build skills for stigma and discrimination reduction and activate change.

**Table 1:** Understanding and challenging drug use stigma in HIV care and treatment clinics curriculum

Session & key topics	Corresponding exercises	Drivers addressed
Session 1: Creating awareness about stigma and introduction to drug use	1) Naming stigma through pictures 2) Stigma-reflection 3) Naming drugs 4) Classification of drugs	• Lack of awareness of stigma • Lack of knowledge of drug use
Session 2: Understanding and addressing fear of interacting with people who use drugs living with HIV	1) What do people say, fear and do about people who use drugs? 2) Why do people start using drugs? 3) Major concerns health workers have about providing services to PWUD who are living with HIV	• Stereotypes • Fear • Lack of knowledge of drug use
Session 3: Understanding drug use, addiction and co-occurring conditions	1) Addiction 2) Physical and psychological dependency 3) Providing treatment and care for clients who use drugs living with HIV	• Lack of knowledge about drug use as a medical condition and absence of skills to care for PWUD
Session 4: Understanding stigma	1) Panel Discussion: Experience-sharing from PWUD 2) Forms, effects and causes of drug use stigma (Problem tree)	• All drivers
Session 5: Working to create change	1) Be the Change! How to provide non-stigmatizing services to people who use drugs 2) Finding solutions to challenge drug use stigma, writing a code of practice	• All drivers

#### Driver 1: lack of awareness of stigma

**Fig. 1:**
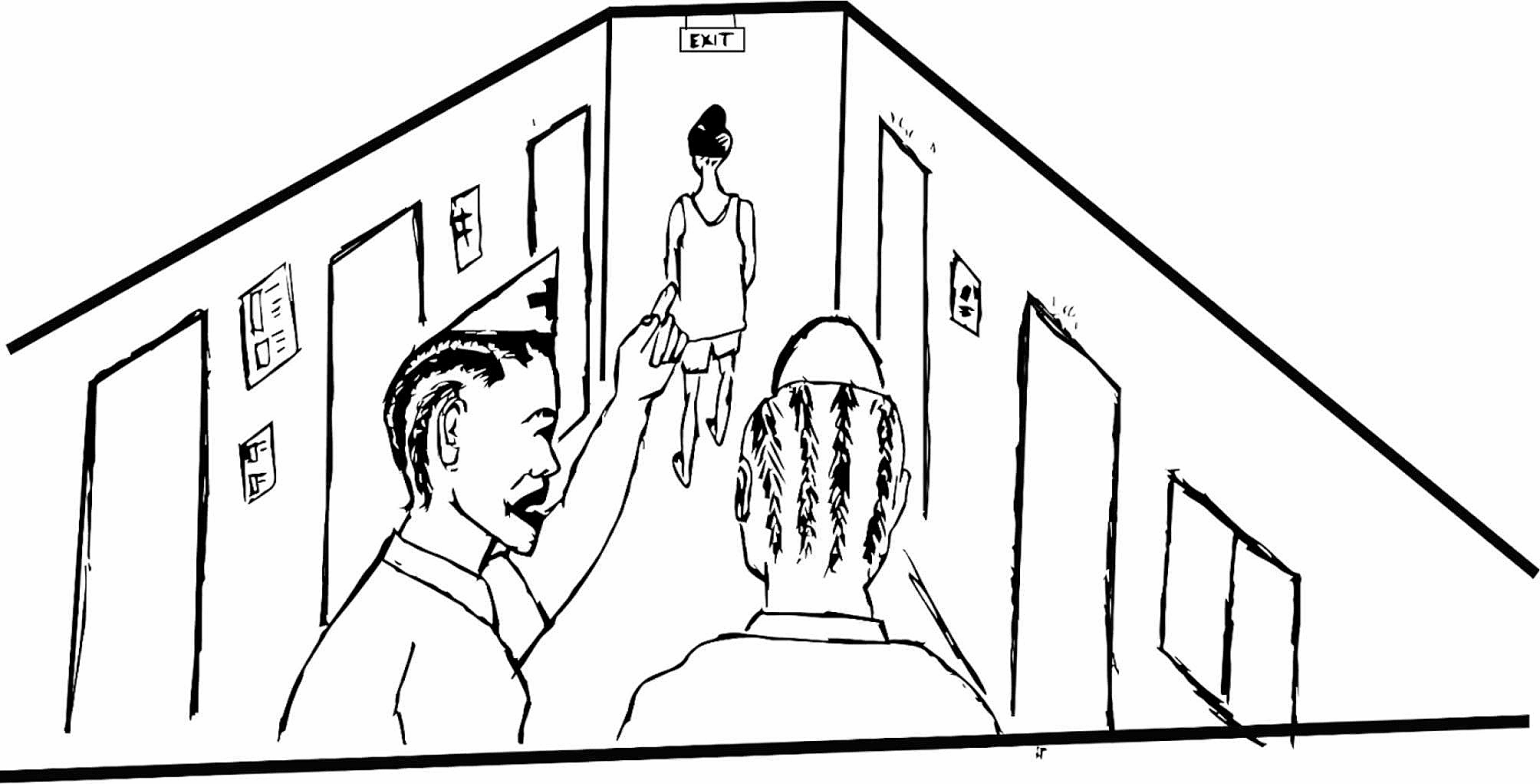
Health care workers gossiping about a person who uses drugs and pointing fingers at them

In most cases, health workers do not intend to stigmatize, they are simply unaware that they are doing so. To address this lack of awareness of how drug use manifests in the clinic, as well as the community, we adapted several existing exercises to include more focus on drug use stigma. For example, in the first exercise in session 1 pictures are used as a tool to generate discussion and recognition of stigma. To adapt this exercise a local artist was commissioned to draw several new pictures which show drug use stigmatizing actions in both community and health settings. For example, a picture showing health care workers gossiping about a person who uses drugs and pointing fingers at them (See Fig. 1). Both are manifestations of stigma that were described in the formative research. Case studies and role plays used in several exercises through all the sessions illustrate specific examples of stigma and its effect. A ‘stigma tree’ exercise deepens understanding of the connections between root causes of stigma, manifestations of stigma that ‘grow’ from those root causes and the effects of the stigma manifestations. The facilitators lead participants to discuss and respond to the following questions in pairs, writing one answer per card which they place at the appropriate place on the tree: Why do people stigmatize? (The roots of the tree); What do people do when they stigmatize? (Manifestations of stigma placed on the trunk of the tree); How does that affect the person stigmatized? (The leaves and branches of the tree).

#### Drivers 2 and 3: negative stereotypes and fear of providing services to PWUD

We addressed the two drivers of stereotypes and fear of providing services together in multiple exercises and across sessions because they are closely linked to each other, with stereotypes (e.g., all PWUD are aggressive) often driving fear (I will be attacked). In Session 2, exercise one, we adapted the HP + HIV stigma exercise around name calling and harmful words and added two additional pieces to it. This exercise employs a rotational small group brainstorm in which each group starts at a separate flip chart that has one of the following questions written on it: *(i) What does the community think or say about people who use drugs?* (Language is a means of communication. Hence, the way one communicates can demonstrates his/her beliefs, attitudes, stereotypes etc. This question aimed to examine the stereotypes that participants had about PWUD) *(ii) How does the community treat people who use drugs? (iii) What does the community fear about people who use drugs? (iv) What is the difference between you and me, and someone who uses drugs? (v) What are the similarities between you, me and someone who uses drugs?* After a few minutes of discussion and writing responses on their respective flip charts, the groups move on to the next flip chart, until they have rotated around the room to all five flip charts. The trainer then facilitates a large group discussion about the responses. The objective is that by the end of this session participants will be able to name their thoughts and feelings about people who use drugs, including their fears, understand more about the stereotypes, attitudes and behaviours directed towards people who use drugs, and reflect on how they might have similarities to people who use drugs.

The specific fears that health workers have about providing services for PWUD are addressed directly in exercise 3 of Session 2. In the first part of this exercise participants discuss in small groups the question, *What are some of the concerns that health facility staff might have about providing services to people who use drugs at the HIV care and treatment clinic (CTC)?* After a full group debriefing of the discussions, participants return to small groups to discuss the following questions: (1) *Where does the fear come from?/ What lies behind the fear? (2) How do clinic staff currently address or deal with the fear? (3) What should be done in the future to deal with the fear?* Each small group then shares their responses with the full group and a list of responses is compiled. The trainer then summarizes the session focusing on how the fear of caring for people who use drugs is a major source of stigma and on the solutions the groups have brainstormed for addressing that fear, leading a discussion on how these can reduce stigma towards clients who use drugs.

#### Driver 4: lack of knowledge about drug use as a medical condition and absence of skills to care for PWUD

We begin building understanding of drug use from the first session (exercise 3 and 4) with two newly developed exercises which inform participants about the various types, names and classification of drugs and their mode of action on the brain, including both illegal drugs such as heroin and cannabis and legal ones such as caffeine, alcohol or nicotine. This exercise builds understanding of the different kinds of substances and, how they act on the brain. It also builds empathy and awareness that most of us consume substances that we might not think of as drugs but that can affect the brain.

Session 2, exercise 2 builds understanding around reasons why people begin using drugs, continuing to build empathy for PWUD. Participants reflect in pairs on the question, 1)*What are some of the reasons people might start using drugs?* Participants then work in groups to read and discuss one of several case studies, responding to questions including: 2*) What new things have you learned about people who use drugs from this case study? 3) Could similar things happen in your family, to your children, colleagues at work, neighbours, or members of your community?*

Session 3 focuses on understanding drug use as a health condition and building comfort with providing services to clients who use drugs. It provides basic information on substance use disorder and its treatment, as well as co-occurring conditions and implications for treatment. In exercise one, participants discuss the meaning of the word addiction and examine radiological images of a healthy and then a diseased heart and a healthy brain and a brain affected by substance use. The aim is for participants to understand that addiction is a brain disease.

In exercise two, understanding of drug use as a health condition is deepened by helping participants understand both the physical and psychological dependency that goes along with the physical changes in the brain explained in exercise 1 of Session 3. To help participants understand physical dependency, exercise 2 starts with a guided fantasy about being ill with flu and imagining the type of care you would want to receive when feeling so ill. The exercise then explains, using pictures, how a healthy brain can change with prolonged drug use and how at a point the ‘brain switch’ that allows people to stop using drugs can fail, resulting in addiction. As well as how behaviors can alter over time with increasing drug use, explaining how a person can become increasingly focused on drug use and less focused on the regular daily activities in their lives as the need for drugs begins pushing out everything else. This exercise ends with a more detailed examination of the symptoms of opioid withdrawal. The second part of this exercise focuses on the psychological impact of addiction, using a story to generate discussion and understanding of cravings and relapses.

After building a basic understanding of drug use, the last exercise in Session 3 turns to address the lack of comfort and skills to care for clients through a series of mini exercises designed to provide participants with some basic knowledge on key considerations for providing care for people who use drugs that may come into the CTC. Specifically: (1) Commonly co-occurring conditions that PWUD living with HIV may have (e.g., TB, some mental health conditions, and Hepatitis B&C); (2) The risk and reasons for either a missed diagnosis or a misdiagnosis of a co-occurring condition; (3) Where there is a potential for drug interactions for co-occurring conditions. These are followed by a brief mini lecture on MAT.

#### Moving to action

The final two Sessions (4 and 5) of the curriculum focus on solidifying the awareness of stigma, how stereotypes and fear lead to stigma, building skills to challenge stigma when it happens and plan for action. To begin session 4, people with drug use experience are invited to share their experiences with the training participants in the form of a talk show style question and answer session. The panel responds to pre-submitted questions from participants and prepared ‘standard’ questions including: *Can you tell us about a time when you have felt stigmatized at a health facility, or if not you, have you heard about someone else’s experience? Can you tell us about a positive experience in a health facility? What did the staff do to make the experience good? Do you have any tips for us (health care workers) on how we can make our services more welcoming?* The panel provides the opportunity for clinic staff to hear firsthand experiences from people who have used drugs and build a connection outside the clinical setting, continuing to build empathy. It also allows participants to connect what they have learned about drug use, craving, withdrawal and treatment options with the experience of the panel members.

The final session of the training starts with a role-playing exercise where participants practice assertively challenging stigma through different clinic scenarios. It then moves to developing a code of practice and action plan, focusing both on what they as individual health facility staff can do and what requires collective action or involvement of management, with responsibility assigned for each action that has been included in the action plan.

## Discussion

The dual burdens of substance use disorders and HIV pose a challenge to national responses to HIV. At the same time that drug use is projected to increase across the globe, including by 40% in Africa by 2030 [1], multiple behavioral, social and structural factors put PWUD at higher risk of HIV acquisition and also pose challenges to utilization of HIV prevention and treatment services. In Tanzania, HIV prevalence among PWUD is 4–7 times higher than in the general population [3]. Stigma towards PWUD, including in HIV care and treatment clinics, has been identified as one of the key barriers to HIV service utilization by PWUD in Tanzania [7, 39, 40]. The importance of developing and implementing interventions to address stigma, including in health services, is underscored in the government’s most recent Health Sector HIV Strategic Plan V (2021–2026) [41]. However, examples of effective drug-use stigma reduction interventions are few, particularly for the sub-Saharan African context. This paper set out a process for adapting an evidence-based HIV stigma reduction intervention to address drug use stigma in HIV clinics, beginning with formative research to elucidate both the drivers and manifestations of stigma and discrimination towards people who use drugs in Tanzania, in the community and within health care facilities, including in HIV care and treatment clinics.

Common manifestations of drug use stigma in health facilities that emerged from the qualitative data and reflect the two stigma drivers of stereotypes about and fear of people who use drugs are consistent with other studies which highlighted negative attitudes of health care workers towards PWUD [22], name calling [60], ignoring PWUD [61], substandard treatment [28], and denial of care [61, 62]. The assumption that most if not all clients who use drugs are dangerous and manipulative and therefore are to be feared has also been described in studies with health workers in the U.S. and U.K. [63, 64]. What appears as a novel manifestation in our study is the common description of HFS taking stigmatizing precautions around PWUD, for example HFS hiding valuables or guards following PWUD to “prevent them from stealing.” These stereotypes and fears which drive stigmatizing behaviors by HFS may pose a significant barrier to PWUD receiving appropriate and compassionate treatment at the health facility, which in turn can undermine utilization of HIV prevention and treatment services such as HIV testing, pre-exposure prophylaxis (PrEP) and ART initiation by PWUD, as well as retention in care.

The lack of knowledge about substance use as a medical condition and attendant discomfort with providing care to clients who use drugs in HIV clinics was another driver that our adapted curriculum responded to and is also identified in the literature as driving stigma. A systematic literature review highlighted lack of knowledge about substance use disorders and lack of skills on managing PWUD as common factors behind health provider stigma [22]. Gilchrist et al. found that professionals who were trained in addiction services held higher opinions of patients with substance use disorders [65].

One driver of drug use stigma in HIV care and treatment clinics that emerged as important to address—lack of awareness of stigma and its consequences—appears to be unique in the drug use stigma literature, but has been documented as a driver for HIV stigma [66–68]. Although health care workers are sometimes unaware of doing actions which are stigmatizing, the effects of those actions on clients are equally harmful, having similarly negative effects as those stigmatizing actions which are intentional. It has also been observed previously that subtle stigmatizing expressions voiced by health care workers are a barrier to recovery among people with mental illness [69].

A key strategy for addressing all four drivers of drug use stigma and associated manifestations in HIV clinics identified in the formative research was incorporating contact strategies into the curriculum, specifically through the panel session with people with lived experienced of using drugs. Other studies have shown that increased contact with PWUD led to lower stigmatizing attitudes [70, 71].

The ADAPT-ITT approach [48] that guided the adaptation of the HP + HIV stigma-reduction curriculum to address drug use stigma in CTCs is well established, including to adapt HIV stigma reduction to address related and intersecting stigmas [47]. For example, Nyblade et al. [47] used the ADAPT-ITT model to guide adaptation of the HP + HIV intervention to address health facility stigma towards gay, bisexual and other men who have sex with men in Ghana. ADAPT-ITT was also used to modify the HIV/AIDS Self-Management Education Program, a stigma-reduction intervention implemented in South Africa for use with Thai men who have sex with men living with HIV [72]. While the specific formative research findings and final adapted drug use stigma curriculum from this study may not be directly generalizable to other contexts, the process and importance of understanding the drivers and manifestations of the specific stigma to make the adaptation contextually relevant is transferrable and could be used in HIV clinics in other settings with the aim to improve the provision and access of various HIV services including HIV testing, PrEP, initiation of ART and its retention leading to HIV viral suppression. As is the importance of the involvement of multiple stakeholders throughout the multiple iterations of the adaptation process, who provided invaluable input, enriched the curriculum, ensured content was relevant, engaging and fully participatory. A context-specific driven limitation to this intervention adaptation process that should be considered in other settings, particularly where polysubstance use is common, is understanding differences in HFS stigma towards use of different substances and the people who use them and how that could shape the drug use stigma-reduction intervention adaptation process. As the majority of drug use linked to HIV in Tanzania is of heroin, PWUD participants in the formative work were either current heroin users or clients of the MAT clinic, so we did not have formative data describing differences in stigma toward different drugs or people who use different drugs.

## Conclusion

Understanding context specific drivers and manifestations of stigma from the perspective of PWUD and health workers providing HIV services in HIV clinics in Tanzania, allowed for ready adaptation of an existing evidence-based HIV stigma reduction intervention. This intervention adaptation process is integral to harm reduction towards PWUD and could serve as an example for other interventions to address drug use stigma within health facilities including HIV care and treatment clinics, and also could be adapted to provide stigma reduction knowledge and skills targeting other forms of stigma, such as HIV stigma in substance use treatment centers.

## Data Availability

Due to the sensitive nature of this study and the criminalized nature of drug use in Tanzania, transcripts from formative research are not publicly available. Requests for data can be submitted to the corresponding author.

## Abbreviations

ART: antiretroviral Therapy
CBO: community based organization
CTC: HIV care and treatment clinic
HP +: Health Policy Plus
HFS: health facility staff
IDI: in-depth interview
Kimara Peers: Kimara Peer Education and Training Trust
MAT: medically assisted therapy
MUHAS: Muhimbili University of Health and Allied Sciences
NatHREC: National Review Ethics Committee
NIMR: National Institute of Medical Research
PWUD: people who use drugs
RQA: rapid qualitative analysis
